# Systematic Design of Molecularly Imprinted Polymers for Triclosan Using Design of Experiments and Molecular Dynamics Simulations

**DOI:** 10.3390/polym18121459

**Published:** 2026-06-11

**Authors:** Martín Carballo-Pacheco, César Ojeda, Maryam Karimi, Payam Zarrintaj, Mir Mehdi Seyedebrahimi

**Affiliations:** 1 Potsdam Transfer, University of Potsdam, Karl-Liebknecht-Str. 24-25, 14476 Potsdam, Germany; 2 Institute of Mathematics, Faculty of Science, University of Potsdam, Karl-Liebknecht-Str. 24-25, 14476 Potsdam, Germany; 3 E3 LLC., Louisville, KY 40059, USA

**Keywords:** molecularly imprinted polymers, design of experiments, molecular dynamics simulation, controlled release

## Abstract

An optimized method of triclosan MIPs using a Design of Experiments (DOE) strategy was developed. The concentrations of methacrylic acid (MAA, monomer), 2-hydroxyethyl methacrylate (HEMA, co-monomer), and acetonitrile (ACN, solvent) were chosen as the critical parameters for the preparation process since they affect imprinting efficacy, morphological structure, and release profile of the material. A Box–Behnken design was utilized for the evaluation of how these factors influence the imprinting factor (IF). The optimized formulation revealed proper IF value indicating efficient molecular recognition. FTIR analysis validated the presence of acrylate-based bonds in the network structure. In addition, SEM images indicated a porous and aggregated structure of MIPs, which facilitated the accessibility of imprinted cavities. Release kinetics revealed two-phase profiles characterized by a moderate initial stage followed by sustained release up to 48 h. The Korsmeyer–Peppas model represented a better correlation (R^2^ = 0.9754) compared to other kinetic models, implying complex diffusion-controlled release processes. Finally, MD simulations confirmed the experimental findings since MAA exhibited higher binding frequencies with triclosan than HEMA, proving its dominant role in molecular recognition.

## 1. Introduction

The extensive application of antimicrobial compounds such as triclosan (TCS) in various fields has increased its ubiquitous occurrence in the environment as well as in biological organisms, thus increasing concern about the adverse effects that TCS can exert on ecosystems due to its toxicity, bioaccumulation capability, and possible negative impacts [1]. Triclosan is a hydrophobic and chlorinated aromatic compound bearing a phenol group, allowing it to undergo a variety of interactions, ranging from hydrogen bonding to hydrophobic and π-π interactions. The physicochemical characteristics of triclosan make it a very difficult compound to separate selectively from water, and its handling poses many challenges [2,3]. Within the available strategies for the selective recognition and elimination of target compounds, MIPs have been proven to be one of the most interesting materials due to their unique property of generating specific binding sites through polymerization processes with the incorporation of a template molecule. Such materials present enhanced selectivity, chemical robustness, and recyclability when compared to traditional sorbents such as activated carbon or other types of polymers [4]. In addition, MIPs showed superior performance compared to traditional sorbents in TCS-related studies [5].

Molecular imprinted polymers are specially designed polymers containing template shaped cavities that make the polymers have high specificity during drug adsorption and delivery processes. For drug delivery purposes, imprinted polymers generally exhibit much higher drug loading capacity and more prolonged drug releasing times relative to non-imprinted polymers and hydrogels [6]. The important factors that need consideration when formulating the polymers include the type and quantity of the functional monomer such as methacrylic acid (MAA), which contributes to drug selectivity, and a hydrophilic co-monomer such as 2-hydroxyethyl methacrylate (HEMA), which helps in enhancing the polarity of the formed polymeric structure. The nature and amount of the cross-linking agent determine the mechanical strength of the network formed as well as its porosity since trifunctional cross-linkers (such as trimethylolpropane triacrylate or TRIM) lead to rigid and larger particles with relatively more drug specificity than the commonly used bifunctional EGDMA [7]. The type and amount of porogenic solvent contribute to the morphology of the polymeric structures. Porogen can be either aqueous (such as water), organic or mixed for both organic and inorganic solvents [8]. For instance, acetonitrile (ACN) is commonly used in nonaqueous imprinting processes while water/polar solvents may be employed for preparing water-compatible MIPs. The efficiency of the process is determined by the imprinting factor (the ratio between MIP/NIP binding) [9]. Recent research shows imprinting factors within a ~2–5 range and a variety of release patterns (often featuring initial burst followed by diffusion). Molecular imprints form molecular memory sites complementary to the drug molecule in terms of both shape and chemical functional groups [9]. The interaction between the template and monomers during imprinting involves non-covalent binding via hydrogen bonds, electrostatics, and hydrophobic or π-π interactions. Once the template is removed, the binding sites formed have high selectivity toward their templates just like antibodies and enzyme active sites. In the case of MAA (containing a carboxylic group), it readily donates hydrogen bonds to hydrogen bond acceptors present in the drug molecule and holds them firmly in place. A more hydrophilic monomer such as HEMA does not interact as strongly with the drugs but results in increased swelling and accessibility [6,9]. In general, drug release from molecularly imprinted polymers follows a diffusion mechanism; the release of surface-bound drugs (bursts) initially occurs rapidly while diffusion through the polymer network takes place more slowly. In addition to increasing crosslinking or designing stronger binding sites, stimuli-responsive materials can be incorporated into the molecularly imprinted polymers, such as those that release doxorubicin when subjected to acidic and reducing conditions [10].

In this context, methacrylic acid (MAA) is among the most popular functional monomers employed in MIP production. This is attributed to the fact that it creates interactions, which allow for highly specific materials. Nevertheless, MAA systems are characterized by high density and low diffusion of polymers, limiting their performance in terms of adsorption kinetics and desorption. As a solution to the problem, hydrophilic co-monomers have been added to increase the polarity of a polymer, its swelling and accessibility of target molecules. For instance, 2-hydroxyethyl methacrylate (HEMA) is a good candidate for this purpose. Moreover, another important factor influencing MIP performance concerns the influence of the porogen used during MIP synthesis on its functionality, as well as pore structure and particle morphology [8,11]. Porogen-assisted polymerization has already been reported to impact adsorption kinetics and adsorption equilibrium via altering the internal structure of the polymer matrix. In triclosan adsorption systems, fast equilibrium processes and good adsorption capacity are commonly achieved with MIPs with developed porosity and a high surface area.

While some previous research focused on one of the influencing factors, such as specific monomers or porosity, a comprehensive characterization of the main contributing factors to develop efficient MIPs for triclosan is still missing. Most existing MIP systems were obtained based on trial-and-error procedures, which do not provide a full understanding about the mutual impact between monomer composition and functional characteristics of the resulting MIP. Currently there is no study to systematically optimize all three main parameters simultaneously, i.e., functional monomer interactions (MAA), hydrophilicity (HEMA) and the effect of the porogen solvent (ACN). In this study, a Design of Experiments (DOE) method utilizing the Box–Behnken design was applied to explore the impact of the contents of MAA, HEMA, and porogens on the performance of triclosan-imprinted polymers. The correlation among the compositions, morphologies, and performances of MIPs was explored by combining the synthetic process, structural analysis, and the adsorption process. The findings reveal that the optimal formula can provide a high imprinting factor (around 3.0) and release efficiency (around 65–70% after 24 h). This paper presents a systematic strategy for creating MIPs with adjustable adsorption and desorption capabilities, which will facilitate their applications in selective adsorption and controlled release of hydrophobic bioactive molecules like triclosan. Unlike the traditional trial-and-error method or parameter-based investigations, the current study uses the Design of Experiments (DOE) technique together with molecular dynamics (MD) simulations to study the influence of functional monomer (MAA), hydrophilic co-monomer (HEMA), and porogen (ACN) on the properties of triclosan-imprinted polymers. The DOE method allows optimizing the dependent variables of the formulation, while MD simulations help us understand at the molecular level the interaction between the template and monomers. While some previous studies have considered individual aspects of the problem such as monomer choice or porosity, no systematic approach for triclosan-imprinted polymers optimization has been performed yet.

## 2. Materials and Methods

### 2.1. Materials

Triclosan (TCS) was used as the template. Methacrylic acid (MAA) and 2-hydroxyethyl methacrylate (HEMA) were employed as functional and hydrophilic comonomers, respectively. Trimethylolpropane triacrylate (TMPTA) served as the crosslinking agent, and azobisisobutyronitrile (AIBN) was used as the thermal initiator. Acetonitrile (ACN) was used as the porogen. All reagents were of analytical grade, purchased from Sigma, St. Louis, MI, USA and used without further purification.

### 2.2. Experimental Design (DOE)

To effectively explore the influence of significant formulation parameters on the behavior of the molecularly imprinted polymers (MIPs), a response surface method (RSM) was used. Three independent factors were chosen because of their importance in the efficiency of imprinting and structural characteristics of the produced polymers: proportion of the functional monomer (MAA, X_1_), proportion of the hydrophilic comonomer (HEMA, X_2_), and porogen volume (ACN, X_3_). The content of the crosslinker (TMPTA) remained unchanged in all experimental trials. The chosen independent variables were varied using three-level factorial analysis, and the experimental design matrix was built following the principles of the Box–Behnken technique. In such a manner, the influence of independent variables and their interactions on the response could be efficiently explored. As the main response variable, the imprinting factor (IF) was used [12]. The experimental design included 15 runs consisting of 12 edge points and three center-point replicates. The center-point replicates were incorporated to evaluate experimental reproducibility and assess potential curvature in the response surface.

### 2.3. Synthesis of Molecularly Imprinted Polymers

The preparation of MIPs was performed using the bulk free radical polymerization technique. Triclosan was first dissolved in the acetonitrile solvent in the appropriate amount followed by the addition of MAA and HEMA according to the DOE matrix. The cross-linker TMPTA was then added to the above mixture, followed by stirring until it was homogenized in the system. Initiation of the reaction was carried out using AIBN in the amount of 0.030 g and nitrogen gas was passed in the mixture for 10 min to get rid of oxygen. The bulk polymerization and curing was done at 60 °C for 24 h and 70 °C for 2 h respectively. The triclosan was extracted using a mixture of methanol and acetic acid in a ratio of 9:1, *v*/*v*. Extraction was stopped when there was no trace of triclosan found in the solvent by UV-Vis spectroscopy (Shimadzu, San Jose, CA, USA).

### 2.4. Characterization

FTIR analysis was done to determine the presence of the polymer structure and removal of the template. The spectra were taken by observing the peaks related to the functional groups (C=O, O-H) and disappearance of the templates. Scanning electron microscope (SEM) was employed for the analysis of the surface morphology and particle structure. The samples were gold-coated prior to taking pictures. The particle size and morphological details were observed based on the SEM images. The triclosan concentration was measured by UV–Vis spectroscopy. UV–Vis measurements for triclosan adsorption and release studies were performed at a wavelength of 280 nm. Calibration curves were drawn for triclosan concentrations. A linear regression model was established: Y = 0.0154X + 0.193 (R^2^ = 0.9914), where X = concentration and Y = absorbance.

### 2.5. Adsorption and Release Experiments

The batch adsorption experiment was done by adding a known weight of polymer to a solution containing triclosan with a known concentration. After equilibration, the polymer was then separated through centrifugation, after which UV-Vis spectroscopy was done for the analysis of the supernatant. Calculation of the adsorption capacity ($q_{e}$) is represented by:

$$q_{e}=\frac{(C_{0}-C_{e})V}{m}$$

Removal efficiency (%) is calculated as:

$$\%Removal=\frac{C_{0}-C_{e}}{C_{0}}\times 100$$

The imprinting factor (IF) is calculated as:

$$IF=\frac{q_{e,MIP}}{q_{e,NIP}}$$

where $C_{0}\;and\;Ce$ are the initial triclosan concentration (mg L^−1^) and equilibrium triclosan concentration after adsorption (mg L^−1^), respectively. V is the solution volume (L), and m is the mass of the polymer used (g). $q_{e,MIP}\;and\;q_{e,NIP\;}$ are equilibrium adsorption capacity of the molecularly imprinted polymer (mg g^−1^) and equilibrium adsorption capacity of the non-imprinted polymer (mg g^−1^), respectively. For release experiments, triclosan-loaded MIPs were suspended in distilled water at neutral pH (~7.0) under continuous shaking at 100 rpm and room temperature. Samples were collected at predetermined time intervals and analyzed using UV–Vis spectroscopy. Release experiments were performed in triplicate (*n* = 3), and the results are reported as mean ± standard deviation. Cumulative release (%) was computed from the amount of triclosan released as compared to the total amount loaded [13,14].

### 2.6. Molecular Dynamics Simulations

A pre-polymerization molecular dynamics model was performed containing triclosan as the template, methacrylic acid (MAA) as the functional monomer, 2-hydroxyethyl methacrylate (HEMA) as the comonomer, and acetonitrile as the porogen. The simulated mixture contained 1 triclosan molecule, 7 MAA molecules, 2 HEMA molecules, and 100 acetonitrile molecules. The ratios were picked to represent condition 1 (Table 1) with 0.1 g triclosan, 0.2 g MAA and 0.1 g HEMA. Small-molecule structures were generated from SMILES strings using RDKit, with explicit hydrogens added prior to 3D coordinate generation. The SMILES used were triclosan, Clc2cc(Cl)ccc2Oc1ccc(Cl)cc1O; methacrylic acid, CC(C(O)=O)=C; 2-hydroxyethyl methacrylate, O=C(OCCO)\C(=C)C; and acetonitrile, CC#N. Initial 3D conformers were generated with RDKit and were subsequently optimized with the MMFF force field [15,16]. Partial charges were assigned with the AM1-BCC method using the OpenFF toolkit [17,18,19,20]. All molecules were described with the Open Force Field small-molecule force field openff-2.2.1.offxml [19,20,21]. The full simulation system was assembled in OpenMM via the OpenFF toolkit using the parameterized component library [19,20,21]. Acetonitrile was modeled explicitly and parameterized in the same manner as the other small molecules. The initial configuration was generated in a periodic cubic box of a side length of 5.0 nm by random insertion of 1 triclosan, 7 MAA, 2 HEMA, and 100 acetonitrile molecules, using a minimum placement padding of 0.16 nm between molecular bounding spheres.

Molecular dynamics simulations were performed in OpenMM 8.5 [22,23]. A Langevin middle integrator was used with a time step of 1.0 fs, a friction coefficient of 1.0 ps^−1^, and a production temperature of 300 K. Pressure was maintained at 1.0 bar using a Monte Carlo barostat with volume-move attempts every 25 steps. Periodic boundary conditions were applied in all three dimensions. The initial system was energy-minimized for 2000 iterations, followed by a 5 ps equilibration at 50 K, and a 100 ps equilibration at 300 K. The production trajectory was propagated for 30 ns. Coordinates were saved every 100 ps. Because this starting configuration was intentionally loose, the system was allowed to relax under NPT conditions at 300 K and 1 bar. The box contracted rapidly during the early stage of the simulation and then fluctuated around a stable liquid-like density. Across the 30 ns production trajectory, the average density was 0.83 g mL^−1^. The average density over the first 1 ns of the production run was 0.826 g mL^−1^, and the final density was 0.824 g mL^−1^, indicating that no substantial drift occurred during the simulation. The final cubic box length was 2.197 nm.

Trajectory analysis focused on triclosan-centered interaction patterns rather than exact structural microstates. All saved frames were processed using a custom Python (version 3.14.3.) analysis workflow based on MDTraj [24]. The trajectory was first imaged under periodic boundary conditions and aligned on triclosan to remove rigid-body translation and rotation before interaction analysis. Two triclosan interaction regions were defined around its two oxygen-centered sites: a phenolic oxygen site (O1) represented by atoms O1x, C1x, C2x, and C6x, and a bridging ether oxygen site (O2) represented by atoms O2x, C7x, C8x, and C12x. For each frame, binary interaction descriptors were assigned according to whether at least one MAA or HEMA molecule formed a contact with either triclosan site and whether hydrogen-bond geometries were present between triclosan and the monomer species. Contact patterns were defined using site-specific heavy-atom proximity between triclosan and MAA or HEMA. Hydrogen-bond patterns were identified geometrically by evaluating donor–hydrogen–acceptor triplets involving triclosan and monomer oxygen atoms. Each frame was therefore assigned to an interaction-pattern family defined by the set of contact and hydrogen-bond events present in that frame. For each interaction pattern, the number of assigned frames and its fraction of the total trajectory were calculated. Representative structures were selected by constructing a triclosan-centered local feature vector for each frame and identifying the frame nearest the centroid of all frames belonging to the same interaction pattern. The molecular dynamics simulations were visually inspected using ChimeraX [25,26].

## 3. Results and Discussion

We studied using DoE the effect of the concentrations of methacrylic acid (MAA, functional monomer), 2-hydroxyethyl methacrylate (HEMA, hydrophilic comonomer), and acetonitrile (ACN, porogen) for a MIP for triclosan detection.

**Figure 1:**
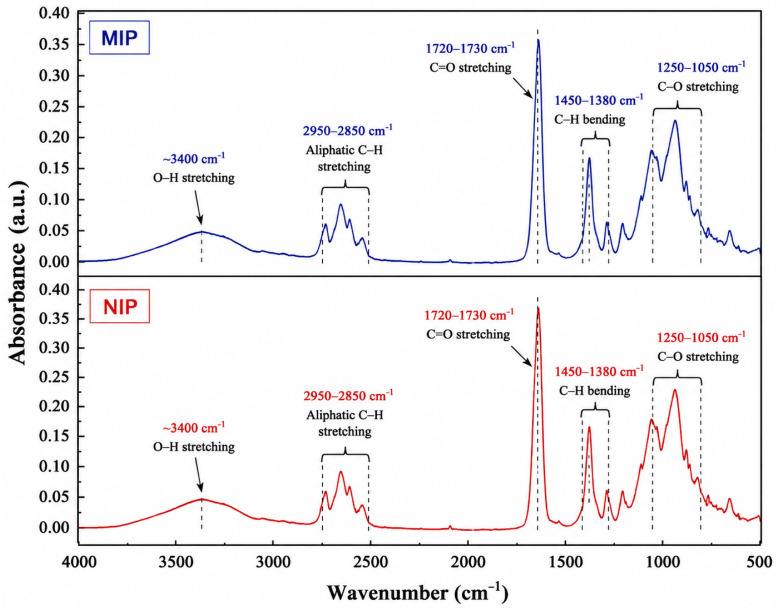
FTIR spectra of the molecularly imprinted polymer (MIP-top) and non-imprinted polymer (NIP). The spectra show the characteristic absorption bands of the acrylate-based polymer network. The broad band around 3400 cm^−1^ corresponds to O–H stretching, the peaks at 2950–2850 cm^−1^ are assigned to aliphatic C–H stretching, and the band at 1720–1730 cm^−1^ corresponds to C=O stretching from methacrylate/carboxyl groups. The presence of these characteristic peaks confirms successful formation of the polymer network, while comparison between MIP and NIP supports the structural similarity of the imprinted and non-imprinted systems.

First, FTIR was employed for confirmation of the acrylate-based polymer network formation and comparison of the chemical structures of the MIP and NIP. As seen in Figure 1 below, the first spectrum belongs to MIP and the second spectrum belongs to NIP. Both of the spectra have absorption bands indicating a polymer network structure, such as a broad O–H stretch in the region of 3400 cm^−1^, aliphatic C–H stretches in the region of 2950–2850 cm^−1^, and a strong C=O stretching band in the region of 1720–1730 cm^−1^ that belong to the methacrylate/carboxyl-containing groups (MAA, HEMA, and TMPTA). It can be concluded that a polymer network was successfully formed in both MIP and NIP. When comparing MIP and NIP, we can see that both types of polymers have the same acrylate-based polymer network. However, some differences in band intensities are seen, possibly because of the effect of the imprinting procedure and the surrounding environment of chemical groups. Additionally, molecular dynamics simulations also show that favorable interactions between triclosan and MAA molecules are formed during complexation [27].

The adsorption capacity of the synthesized MIPs and their non-imprinted analogs towards triclosan were determined using batch rebinding experiments. The results obtained show that MIPs possess greater adsorption capacity than NIPs (see Table 1). This proves that the formation of selective cavities for triclosan molecules took place. The selectivity of MIPs towards triclosan may be explained by the formation of complementary cavities with respect to triclosan molecules which result from the polymerization process. In this regard, NIPs show significantly lower binding capacity due to absence of specific cavities as there are no cavities for specific interaction with target molecules. Thus, nonspecific adsorption due to van der Waals forces occur in this case. On the molecular level, the binding process depends on the formation of complexes between triclosan and methacrylic acid due to bonding between triclosan and carboxyl groups. In addition, the addition of 2-hydroxyethyl methacrylate improves the wettability of polymers, thus increasing the adsorption capacity of the resulting material [28].

**Figure 2:**
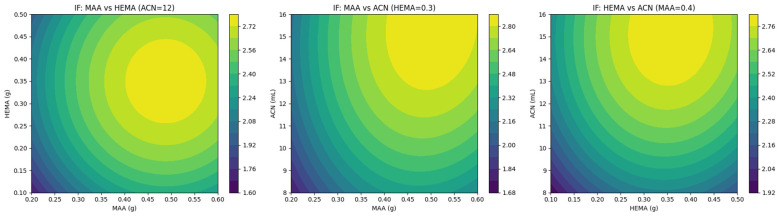
Response surface contour plots showing the effects of MAA, HEMA, and ACN on the imprinting factor (IF) of triclosan-imprinted polymers. MAA represents methacrylic acid, HEMA represents 2-hydroxyethyl methacrylate, and ACN represents acetonitrile used as the porogenic solvent. The contour plots illustrate the interaction effects of formulation variables on molecular imprinting performance. The highest IF values were observed at approximately MAA = 0.45–0.50 g, HEMA = 0.30–0.35 g, and ACN = 14–16 mL, indicating that an optimized balance of functional monomer content, hydrophilicity, and porogen solvent volume improves binding-site formation and accessibility.

We plotted contour plots to understand how the imprinting factor depends greatly on the interaction among MAA, HEMA, and ACN (see Figure 2). From the plot for MAA and HEMA (ACN = 12 mL), the value of IF rises from roughly 1.6 in case of small quantities of MAA (~0.2 g) and HEMA (~0.1 g) to about 2.7–2.8 in case of a high quantity of MAA (≈0.45–0.50 g) and medium amounts of HEMA (≈0.30–0.35 g). This clearly demonstrates that the increase in MAA content contributes to the effective formation of the binding sites because of greater bonding with triclosan, while the presence of medium HEMA leads to increased swelling and better diffusion. In the contour plot of MAA vs. ACN (HEMA = 0.3 g), the IF value rises from ≈1.7 (low ACN ≈ 8) up to ≈2.8–2.9 (high ACN ≈ 14–16 mL and MAA ≈ 0.45–0.50 g), which implies that an increased content of porogen contributes to more efficient pore formation and better access to the binding cavities. From the contour plot of HEMA vs. ACN (MAA = 0.4 g), the IF value reaches ≈ 2.7–2.8 when HEMA ≈0.30–0.35 g and ACN ≈ 14 mL. On the whole, it can be seen from the contour plot that the optimal area corresponds to the composition of MAA = 0.45–0.50 g, HEMA = 0.30–0.35 g, and ACN = 14–16 mL, where the value of IF is about 2.8–2.9, reflecting selectivity of the molecularly imprinted polymers. From these results, it can be inferred that the imprinting factor depends not only on the nature of the polymer structure but also on its functional activity: MAA provides binding specificity, while ACN and HEMA affect the polymer structure through pore size and swellability. The decrease in the IF value with low values of MAA or ACN demonstrates the importance of having enough functional sites and pores. We can say that the DOE results indicate that the imprinting factor was governed by interaction effects among MAA, HEMA, and ACN rather than by a single formulation variable; MAA primarily contributed to triclosan-specific binding-site formation, HEMA improved swelling and diffusion, and ACN enhanced porosity and accessibility of the imprinted cavities. Response surface methodology indicated that the degree of imprinting was influenced by the synergistic effect of the three factors, MAA, HEMA, and ACN, and was not determined independently by each of them. The main function of MAA was related to the formation of recognition sites due to interactions between carboxyl groups in the cross-linkers and triclosan molecules. But when MAA concentration exceeded the optimal value, without proper amount of porogens, a more compact network would form, and recognition sites would be less accessible. Meanwhile, HEMA increased the hydrophilicity of polymers and their ability to swell, making them be able to absorb more triclosan. ACN served as a porogen, helping create pores in the polymer and providing better mass transfer. So, the best formulation was achieved by combining all these aspects. This is how we can explain why the highest imprinting factors were registered in some range of compositions but not at the maximum level of one of these factors.

**Figure 3:**
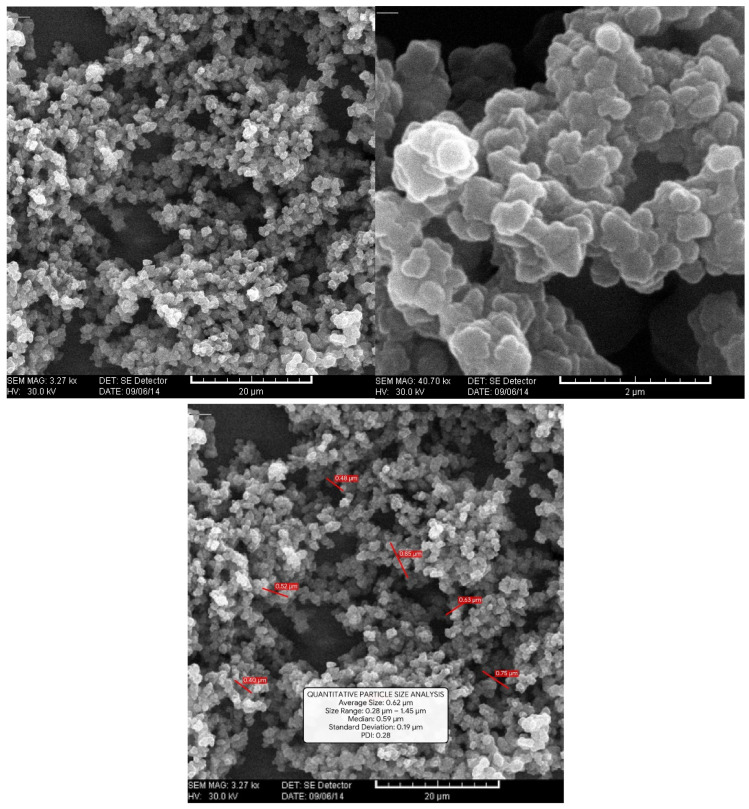
SEM image of the optimized triclosan-imprinted molecularly imprinted polymer (MIP). The micrograph shows the surface morphology of the optimized MIP formulation prepared using MAA, HEMA, TMPTA, and ACN. The polymer exhibits a rough, heterogeneous, aggregated, and porous morphology typical of bulk-polymerized MIP systems. The primary particles are approximately 0.4–1.2 µm in size, with larger agglomerates in the range of 5–25 µm. This porous and aggregated structure may improve triclosan diffusion and enhance access to imprinted binding cavities.

Optimized MIP surface morphologies were studied using SEM analysis. The microphotograph in Figure 3 depicts a heterogeneous, rugged, and very aggregated morphology of optimized MIP, which is typical for bulk MIP systems. The particles form nodular domains that are connected into porous clusters. Using the SEM magnification scale, it was determined that the size of primary particles was within the range of about 0.4–1.2 µm, and an average primary particle size was ~0.8 µm, whereas larger agglomerates had sizes within the range of 5–25 µm. The existence of submicron-sized primary particles means that the MIP polymerization network comprised domains rather than large compact particles, which is common for conventional bulk-type MIP systems. Based on the observation of this surface morphology, it can be stated that this optimized MIP exhibits a porous surface structure, which probably arises from a high content of porogen (ACN). The optimized formulation, corresponding to MAA ≈ 0.50 g, HEMA ≈ 0.35 g, and ACN ≈ 14–16 mL, exhibited a predicted imprinting factor (IF) of approximately 2.8–2.9, consistent with the response surface analysis. The relatively high IF can be directly correlated with the observed morphology. The submicron particle size and porous structure reduce diffusion limitations and allow triclosan molecules to access imprinted cavities more efficiently. The improved accessibility in the present system enables effective utilization of binding sites, resulting in enhanced selectivity and higher IF values. At the same time, the presence of well-defined cavities formed through imprinting ensures that nonspecific adsorption remains limited, maintaining selectivity [29].

**Figure 4:**
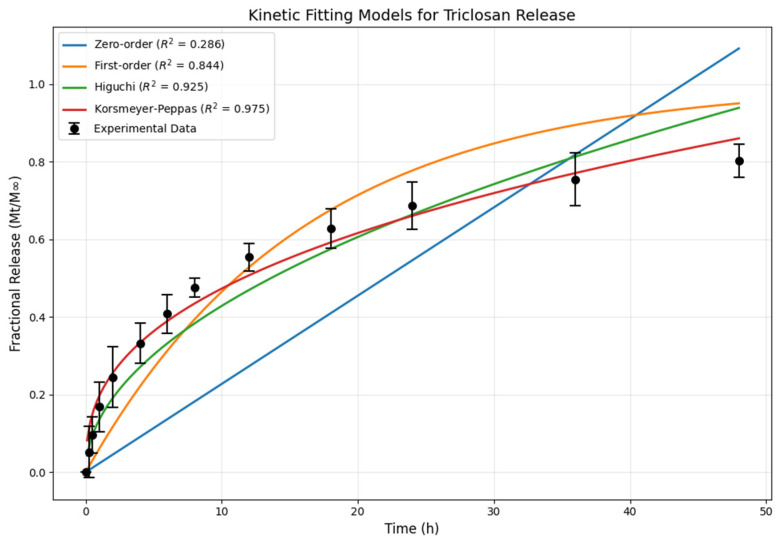
Triclosan release profile from the optimized MIP and fitting with kinetic release models. The fractional release of triclosan, expressed as Mt/M∞, was monitored over 48 h in distilled water at approximately neutral pH under continuous shaking at room temperature. Experimental release data were fitted using zero-order, first-order, Higuchi, and Korsmeyer–Peppas kinetic models. The release profile showed a two-stage pattern, with an initial moderate release followed by sustained release. The Korsmeyer–Peppas model provided the best fit, with K = 0.1968, n = 0.3810, and R^2^ = 0.9754, suggesting a diffusion-controlled release mechanism from the imprinted polymer matrix.

The release behavior of triclosan from the optimized molecularly imprinted polymer (MIP) was evaluated over a 48 h period, and the fractional release (M_t_/M_∞_) is presented in Figure 4. The experimental data show a characteristic two-stage release profile, consisting of an initial moderate release followed by a slower, sustained release phase. In the early stage (0–8 h), approximately 45–50% of triclosan was released, which can be attributed to the diffusion of weakly bound or surface-accessible molecules. This is followed by a slower release phase, reaching approximately 75–80% release at 48 h, indicating the presence of bound triclosan within imprinted cavities. Moderate initial release followed by sustained diffusion-controlled release suggests that the system effectively retains triclosan within the polymer network, consistent with template–monomer interactions.

To elucidate the release mechanism, the experimental data were fitted to four commonly used kinetic models: zero-order, first-order, Higuchi, and Korsmeyer–Peppas models. The fitting results and corresponding correlation coefficients ($R^{2}$) are summarized below (Table 2):

The Korsmeyer–Peppas model provided the best fit to the triclosan release profile, with a release rate constant of K = 0.1968, a release exponent of n = 0.3810, and an R^2^ value of 0.9754, suggesting a diffusion-controlled release mechanism from the polymeric matrix. This model is used when release processes are not ideal and several mechanisms work together. A high correlation coefficient of the Korsmeyer–Peppas model proves that a combination mechanism controls the release from the studied matrix. This mechanism consists of the following steps: desorption from the imprinted sites; interaction of triclosan molecules with functional groups (MAA and HEMA)—breaking of these interactions takes time, hence, slowing down the second stage of release—and diffusion within the polymer matrix—the porous nature of the polymer due to the presence of porogen (ACN) causes gradual diffusion of triclosan molecules. This can be seen by a good fitting of the Higuchi model (R^2^ = 0.9250), which corresponds to diffusion-controlled release from the matrix. Also, a relatively good fit of the first order model (R^2^ = 0.8441) shows that concentration gradient effects take place during the process. Zero-order model (R^2^ = 0.2864) does not correlate with data well, which means that a constant rate release mechanism did not occur in the system [30].

**Figure 5:**
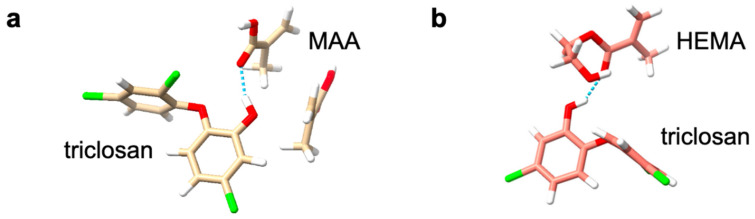
Representative centroid structures of the most common triclosan–monomer contact motifs in the corrected 30 ns trajectory. (**a**) Structure showing MAA contacting both the phenolic hydroxyl and diaryl ether regions of triclosan (15.3% of frames). (**b**) Structure showing HEMA contacting both the phenolic hydroxyl and diaryl ether regions of triclosan (10.3% of frames).

Finally, we perform molecular dynamics simulations to understand the most significant interaction patterns between MAA, HEMA and triclosan. Molecular dynamics simulations can be used to investigate MIP behavior by modeling the dynamic template–monomer–solvent interactions that govern pre-polymerization complex formation, binding-site development, and selective rebinding [31,32,33,34]. Analysis of the trajectory showed that triclosan interacted more frequently with MAA than with HEMA. It should be noted that the simulated mixture contained seven MAA molecules per triclosan, so only a subset of MAA molecules could interact directly with the template at any given time, consistent with a saturable local binding environment around the available triclosan interaction sites. The most populated single state was with no specific interactions between triclosan and the monomers (36% of frames), indicating that the local environment remained dynamic and that persistent binding was not maintained throughout the whole simulation. The most common MAA pattern was simultaneous MAA contact with both the phenolic hydroxyl region and the diaryl ether oxygen region of triclosan (15.3% of frames; Figure 5a). HEMA also interacted substantially with triclosan, with the most common HEMA pattern being simultaneous HEMA contact with both the phenolic hydroxyl region and the diaryl ether oxygen region (10.3% of frames; Figure 5a). It can be said that MAA-only patterns accounted for 31% of frames, compared with 15.7% for HEMA-only patterns, while mixed MAA + HEMA environments accounted for 17.3% of frames. These results indicate that MAA remains the dominant recognition partner for triclosan in this pre-polymerization mixture, but that recognition is governed mainly by dynamic contact-based association around the phenolic and diaryl ether regions of triclosan rather than by persistent specific bonding.

The increased activity of the optimized MIPs could be justified on the basis of both the experimental data obtained during optimization and the molecular insights obtained via MD modeling. It is possible that high MAA content increased the amount of the available functional groups capable of interactions with triclosan molecules, whereas HEMA added the required hydrophilicity, thus facilitating the diffusion process within the polymer network. Furthermore, the solvent amount used in the optimal condition could enhance the formation of the cavities available for binding of the triclosan molecules. Experimental observations agree with those obtained through MD simulations and suggest the occurrence of complexation between triclosan molecules and functional monomers. Hence, optimized compositions exhibit higher binding capacity since there are enough available cavities and favorable interactions between the template and monomers. As for the present study, no experiments on the selectivity were conducted; however, in the future study, the selectivity of the optimized MIPs will be tested against similar compounds, including phenols.

The optimized ration in the DOE model can be attributed to the different functional properties of MAA, HEMA, and ACN in imprinting reactions. MAA is a functional group that contains carboxylic acid groups that can bind triclosan before the polymerization reaction through interaction, thus forming cavities that are recognition sites for the triclosan. However, functional monomers can result in denser polymers with reduced porosity, hindering the diffusion of the drug. HEMA increases hydrophilicity and swelling of the polymer matrix, resulting in better diffusion and access to the cavity for the drug molecules. Similar functionality applies for the addition of ACN since this serves as a porogen, resulting in more pores and facilitating the diffusion of the drug into the cavities. Based on this, the optimal combination includes functional monomers, hydrophilicity, and porosity. In line with this theory, the MD simulations revealed that MAA was more active in binding with triclosan compared to HEMA.

## 4. Conclusions

In the present work, a systematic and integrated approach that utilizes the combination of Design of Experiments (DOE) and molecular dynamics (MD) was successfully applied in the development and optimization of triclosan-imprinted molecularly imprinted polymers (MIPs). Through the utilization of the Box–Behnken design of experiments, the effects of MAA, HEMA, and ACN were investigated simultaneously in regard to their impact on imprinting efficacy. While traditionally, trial-and-error approaches have been used in optimization processes, the application of DOE proved to be an efficient means for identifying the optimal composition and allowed elucidation of the fact that the imprinting factor depended not on individual parameters but rather was determined by synergism. The optimal formulation also displayed an imprinting factor of about 2.8–3.0, thus validating the success of producing the recognition sites for the triclosan molecule.

The FTIR spectra confirmed the formation of the acrylate-based polymer matrix and the structural similarity between the MIP and NIP systems, without compromising the selectivity of the molecularly imprinted one. From the SEM images, a surface structure characterized by porousness and aggregations of submicron primary particles was evident, thus suggesting the positive impact of the optimal porogen amount on the creation of accessible binding sites and minimized diffusion barriers. Release experiments exhibited a typical two-step profile of release where an initial moderate release was followed by a diffusion-controlled phase lasting up to 48 h. Based on kinetic studies, the best fit with the experimental data was obtained for the Korsmeyer–Peppas model, which indicated that the release of triclosan occurs via diffusion through the polymer matrix as well as desorption from the imprinted binding sites. Such a controlled release profile proves the possibility of developing MIP-based systems for controlled release of hydrophobic bioactive molecules. One advantage of the current research is the combination of modeling and experimentation. The molecular dynamics study gave insights at the molecular level on the pre-polymerization interaction and confirmed that MAA forms more favorable contacts with the template than HEMA. This explains the dominating role played by MAA in the imprinting process and justifies the finding that the increase in MAA content leads to a better imprinting result provided the proper amounts of HEMA and ACN are used. It can be clearly seen that the results of the DOE and the molecular simulation correlate well, which means that imprinting efficiency depends on molecular recognition, polymer accessibility, and pore structure.

Finally, this study presents a scientific approach to molecularly imprinted polymer development through the combination of experimental design and molecular modeling. Not only does this approach improve the knowledge about the processes associated with the synthesis of imprinted polymers, but it also suggests an approach that can be used for further development of MIPs aimed at different pollutants, medicinal drugs, and biomolecules. Future studies can include research on selective binding, competitive adsorption of the analyte from solution, as well as scaling up the results obtained to industrial-level production of MIP-based products.

## Figures and Tables

**Table 1:** Design matrix used for optimization of triclosan-imprinted molecularly imprinted polymers (MIPs). The table presents the experimental runs generated by varying methacrylic acid (MAA, functional monomer), 2-hydroxyethyl methacrylate (HEMA, hydrophilic comonomer), and acetonitrile (ACN, porogen). The imprinting factor (IF) was used as the response variable to evaluate the molecular recognition efficiency of each formulation. Higher IF values indicate improved selective recognition of triclosan by the imprinted polymer network.

Run	MAA (g)	HEMA (g)	ACN (mL)	IF
1	0.2	0.1	12	1.6
2	0.5	0.1	12	2.3
3	0.2	0.4	12	1.85
4	0.5	0.4	12	2.65
5	0.2	0.25	8	1.55
6	0.5	0.25	8	2.25
7	0.2	0.25	16	1.95
8	0.5	0.25	16	2.85
9	0.35	0.1	8	1.75
10	0.35	0.4	8	2.05
11	0.35	0.1	16	2.1
12	0.35	0.4	16	2.75
13	0.35	0.25	12	2.7
14	0.35	0.25	12	2.75
15	0.35	0.25	12	2.72

**Table 2:** Kinetic models used to evaluate triclosan release from the optimized molecularly imprinted polymer (MIP).

Model	Equation	R^2^	Interpretation
Korsmeyer–Peppas	Q = K.t^n^	0.9754	Best fit; describes complex diffusion.
Higuchi	Q = K.t^0.5^	0.925	High fit; indicates matrix-based diffusion.
First-order	Ln(1 − Q) = −K.t	0.8441	Moderate fit; release depends on concentration.
Zero-order	Q = K.t	0.2864	Poor fit; release is not at a constant rate.

## Data Availability

The original contributions presented in this study are included in the article/Supplementary Materials. Further inquiries can be directed to the corresponding authors.

## Supplementary Materials

The following supporting information can be downloaded at: https://www.mdpi.com/article/10.3390/polym18121459/s1, Table S1. Comparative summary of triclosan release studies reported in the literature, including polymer/material systems, release conditions, release behavior, kinetic models, and key performance outcomes in comparison with the developed MIP formulation.
