# Supplementary material for: Systematic Design of Molecularly Imprinted Polymers for Triclosan Using Design of Experiments and Molecular Dynamics Simulations
Source: Polymers (Basel). 2026 Jun 11;18(12):1459. doi: 10.3390/polym18121459 (PMC13306850; doi:10.3390/polym18121459)
Supplement: Supplementary file 1 [file polymers-18-01459-s001.zip › polymers-4320310-supplementary.pdf]

Table S1. Comparative summary of triclosan release studies reported in the literature, including polymer/material systems, release conditions, release behavior, kinetic models, and key performance outcomes in comparison with the developed MIP formulation.

Study	Triclosan Delivery System	Polymer/Carrier Composition	Application	Release Condition	Main Release Finding	Release Mechanism/Model	Comparison with Present Study
Kockisch et al. [1]	Mucoadhesive microspheres	Gantrez™ MS-955, Carbopol™ 974P, polycarbophil, or chitosan	Oral-care/dental paste	Simulated oral “in-use” conditions	Triclosan was rapidly released from most microspheres, while chitosan microspheres showed more sustained release; release from non-aqueous paste was sustained and polymer-dependent.	Zero-order behavior for Gantrez, Carbopol, and polycarbophil microspheres; Baker–Lonsdale model for chitosan microspheres.	Demonstrates polymer-dependent release but does not include molecular imprinting or template-specific recognition.
Steinberg et al. [2]	Local sustained-release dental device	Ethylcellulose-based nondegradable matrix and degradable methacrylate ester matrix	Treatment of *Streptococcus mutans* biofilm	In vitro oral/biofilm-related testing	Ethylcellulose device released about 80% of loaded triclosan over 10 days; methacrylate ester matrix provided faster release.	Ethylcellulose system followed Higuchi diffusion behavior; degradable methacrylate system was erosion-controlled.	Provides longer release duration, but the design is based on diffusion/erosion rather than imprinted recognition sites.
Chung et al. [3]	Antimicrobial coating	Styrene-acrylate copolymer containing triclosan	Antimicrobial food packaging	Water, 10% ethanol, and n-heptane food simulants	Triclosan was not released into water, only a very small amount was released into 10% ethanol, while most triclosan was extracted into n-heptane.	Partition-controlled release/extraction depending on medium polarity.	Shows strong medium-dependence; unlike the present MIP, this system is a passive antimicrobial coating rather than a selective drug-delivery matrix.

Iconomopoulou & Voyiatzis [4]	Biocide-loaded polymer films	Triclosan-loaded high-density polyethylene films	Controlled antimicrobial release	Polymer films drawn at different ratios	Molecular orientation of the polymer matrix was proposed as an important parameter controlling triclosan release.	Diffusion affected by polymer orientation and matrix structure.	Highlights the role of physical polymer structure; the present work instead tunes release through formulation variables and imprinting-site accessibility.
Liauw et al. [5]	Silicone elastomer/clay composite	PDMS containing organically modified montmorillonite and triclosan	Antimicrobial elastomer	Leaching study	Organically modified montmorillonite controlled triclosan release from silicone elastomer; Cloisite® 15A gave the most sustained release.	Release governed by triclosan–clay–PDMS interactions and intercalation behavior.	Uses inorganic clay reservoirs to slow release; the present MIP uses polymeric recognition cavities for selective adsorption/desorption.
Present study	Molecularly imprinted polymer	Triclosan-imprinted MAA/HEMA/TMPTA network prepared using ACN as porogenic solvent	Selective adsorption and controlled release of triclosan	In vitro release medium used in this study	Optimized MIP showed controlled triclosan release with reduced burst behavior and sustained release over the studied period.	Best described by Korsmeyer–Peppas behavior, indicating complex/anomalous transport involving diffusion, polymer network effects, and triclosan–binding-site interactions.	Unlike many previous triclosan carriers, the present system combines controlled release with molecular imprinting, enabling selective triclosan recognition and tunable release behavior.

[1]. Kockisch, S., Rees, G. D., Tsibouklis, J., & Smart, J. D. (2005). Mucoadhesive, triclosan-loaded polymer microspheres for application to the oral cavity: preparation and controlled release characteristics. European Journal of Pharmaceutics and Biopharmaceutics, 59(1), 207-216.

[2]. Steinberg, D., Tal, T., & Friedman, M. (2006). Sustained-release delivery systems of triclosan for treatment of Streptococcus mutans biofilm. Journal of Biomedical Materials Research Part B: Applied Biomaterials: An Official Journal of The Society for Biomaterials, The Japanese Society for Biomaterials, and The Australian Society for Biomaterials and the Korean Society for Biomaterials, 77(2), 282-286.
[3]. Chung, D., Papadakis, S. E., & Yam, K. L. (2003). Evaluation of a polymer coating containing triclosan as the antimicrobial layer for packaging materials. International Journal of Food Science and Technology, 38(2), 165-169.
[4]. Iconomopoulou, S. M., & Voyiatzis, G. A. (2005). The effect of the molecular orientation on the release of antimicrobial substances from uniaxially drawn polymer matrixes. Journal of controlled release, 103(2), 451-464.
[5]. Liauw, C. M., Taylor, R. L., Maryan, C., Kato, R., Wilkinson, A. N., & Cheerarot, O. (2011, March). Organo-Montmorillonite as a Controlled Release Reservoir for Triclosan in Silicone Elastomer: A Flow Micro-Calorimetry and Leaching Study. In Macromolecular Symposia (Vol. 301, No. 1, pp. 96-103). Weinheim: WILEY-VCH Verlag.
